# Comparative synthesis and characterization of nanocomposites using chemical and green approaches including a comparison study on *in vivo* and *in vitro* biological properties

**DOI:** 10.1039/d2na00677d

**Published:** 2022-12-13

**Authors:** Sabeena G, Vainath Praveen S, Pushpalakshmi E, Annadurai G

**Affiliations:** a Sri Paramakalyani Centre of Excellence in Environmental Sciences, Manonmaniam Sundaranar University Alwarkurichi – 627412 India gannadurai@msuniv.ac.in annananoteam@gmail.com

## Abstract

In this study, the anti-diabetic, anti-inflammatory, anti-cytotoxic, and antibacterial effects of various substances were studied *in vitro*. Malachite green's photocatalytic effects were used to determine the optimised sample while it was exposed to visible light. The intended nanocomposites were created without any contaminants, according to XRD data. The overall characterisation results of the green synthesis of CS/SiO_2_/TiO_2_/CeO_2_/Fe_3_O_4_ nanocomposites (CSTCF(G)) were superior to those of the chemical synthesis of CS/SiO_2_/TiO_2_/CeO_2_/Fe_3_O_4_ nanocomposites (CSTCF(C)). At the five doses examined, the green synthesis of CS/SiO_2_/TiO_2_/CeO_2_/Fe_3_O_4_ nanocomposites (CSTCF(G)) and chemical synthesis of CS/SiO_2_/TiO_2_/CeO_2_/Fe_3_O_4_ nanocomposites (CSTCF(C)) resulted in higher α-glucosidase inhibition percentages in the antidiabetic assay. HaCaT cells and MCF-7 cells were less harmful when treated with chemically synthesized CS/SiO_2_/TiO_2_/CeO_2_/Fe_3_O_4_ nanocomposites (CSTCF(C)), and green synthesized CS/SiO_2_/TiO_2_/CeO_2_/Fe_3_O_4_ nanocomposites (CSTCF(G)). From the results of the cytotoxicity tests against MCF-7 cells and HaCaT cells using the nanocomposites, the IC_50_ values of *Salacia reticulata*, green synthesized CS/SiO_2_/TiO_2_/CeO_2_/Fe_3_O_4_ nanocomposites (CSTCF(G)), and chemically synthesized CS/SiO_2_/TiO_2_/CeO_2_/Fe_3_O_4_ nanocomposites (CSTCF(C)) were calculated. This research work shows that the green synthesized CS/SiO_2_/TiO_2_/CeO_2_/Fe_3_O_4_ nanocomposites (CSTCF(G)) have strong anti-inflammatory, antibacterial and anti-diabetic properties, as well as considerable suppression of high activation in *in vivo* zebrafish embryo toxicity. The novelty of this study focused on the revelation that green synthesized nanocomposites are more affordable, environmentally friendly and biocompatible than chemically synthesized ones.

## Abstract 2

In this study, the anti-diabetic, anti-inflammatory, anti-cytotoxic, and antibacterial effects of various substances were studied *in vitro*.

## Graphical abstract

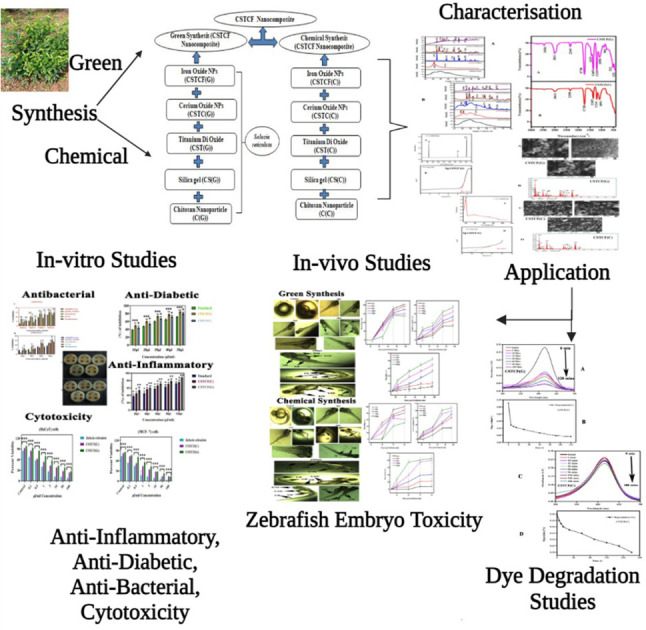

## 1. Introduction

Nanotechnology has sustainable uses in the bio-pharma, food, leather and textile sectors. Research on the green and chemical synthesis of organic coated inorganic nanoparticles is an emerging topic in nanoscience and nanotechnology.^1,90–93^

Chitosan (CS) is a natural biopolymer generated from chitin deacetylation that possesses unique functional features such as biocompatibility, good surface binding capabilities, biodegradability, and antibacterial capabilities. Chitosan nanocomplexes provide a new class of nanocomplexes with improved characteristics and applications.^2–6^

Cerium is a rare earth metal that belongs to the lanthanides (atomic number = 58). The localisation of the cerium 4f electrons facilitates the production of the Ce^3+^/Ce^4+^ redox pair. The ceria catalytic activity involves surface oxygen vacancies and the presence of Ce^3+^ at defect sites.^7–9^ Nanoceria is used commercially in cosmetics, consumer items, instrumentation and high-tech industries. Biomedical applications include the ability to protect cells from radiation, toxicant-mediated cell damage, and pathological conditions such as brain or heart ischemia, neurological disorders and retina neurodegeneration.^10,11^ The literature on the toxicity of CeO_2_ has contradictory conclusions. Some research provides an in-depth examination of the anticancer effects of different materials containing CeO_2_ nanoparticles.^12–15^

In nature, iron oxides exist in a variety of forms, with magnetite (Fe_3_O_4_), hematite (α-Fe_2_O_3_), and maghemite (γ-Fe_2_O_3_) being the most frequent and important technologically.^16^ Surface effects have been shown to have a significant impact on the magnetic properties of iron oxide nanoparticles (NPs).^17^ Because of this, there is a need to synthesize such absorbents with suitable particles sizes for the removal of heavy dyes from industrial waste dye and organic dyes.^18^

With reference to the various applications of NPs, the anti-cancer properties of these materials are appealing in the treatment of several tumours, because the use of NPs has been linked to anti-cancer effects against a number of cancers.^19–21^ Some semiconductor nanoparticles, such as ZnO, Dy_2_Ti_2_O_7_, CaWO_4_, CdTiO_3_, NdVO_4_, and TiO_2_, are now being explored as photocatalysis materials for the clean up of polluted water.^22–27^ However, prior research reveals that bare TiO_2_ based-nanostructured photocatalysts have poor photocatalytic performance under visible light, can experience charge carrier recombination, and have a narrow light-response range, which would justify delaying their use in photocatalytic applications.^28^

From previous related work, a layer, such as a controlled silicon oxide layer, between a magnetic core and photocatalyst shell can reduce the negative effect of iron oxide on the titanium oxide photocatalysis process, retain the magnetic characteristics, shield Fe_3_O_4_ from oxidation, and improve the removal efficiency. Several studies have recently been conducted on the development of recyclable photocatalytic nanocomposites of Fe_3_O_4_/SiO_2_/TiO_2_ with a core–shell structure.^29^

In earlier work on the magnetic separation of Fe_3_O_4_/TiO_2_ utilising an external electromagnetic field, magnetic composites are recycled after the dye degradation process. The coupling of Fe_3_O_4_ magnetic nanoparticles to TiO_2_ and CeO_2_ photocatalytic nanoparticles has the advantages of providing a unique magnetic response, a chemically changeable surface, and environmental benignity.^30,31^ Furthermore, a TiO_2_ coating of Fe_3_O_4_ NPs hinders their large accumulation. Additionally, solitary Fe_3_O_4_ nanoparticles are vulnerable and unstable under the reaction conditions, and the interaction of Fe_3_O_4_ nanoparticles with TiO_2_ nanoparticles induces the recombination of electrons and holes, lowering the photocatalytic capability.^32,33^

Since ancient times, *Salacia reticulata*, also known as “Ponkoranti” in Tamil, has been utilised in Ayurveda to cure diabetes. Because of its anti-diabetic properties, it can prevent and restrain an enzyme from combining glucose in the intestinal wall. It is used in the making of herbal teas for diabetes. The plants have qualities that are stimulating, laxative, diuretic, cardiotonic, anthelmintic, and anti-diabetic.^86^*Salacia reticulata* contains phenolics, alkaloids, flavonoids, saponins, tannins, steroids, glycosides/reducing sugars, glycosides/cardiac glycosides, phlobatannins, and other secondary metabolites as phytochemicals.^87,88^

In contrast to nanocomposites made using conventional techniques, there is debate regarding whether green synthesis is environmentally beneficial and whether the nanocomposites created in this way are biocompatible.

In this study, our research team successfully combined the advantages of heterogeneous catalysis with green and chemically synthesised CSTCF(G) and CSTCF(C) nanocomposites, followed by the further application of the *in vivo* and *in vitro* biological properties. Similarly, the prepared green and chemically produced materials were used in the photocatalytic degradation of malachite green (MG) dye under UV light irradiation. The novelty of the study focused on the identification of any potential differences between the properties, yields, and toxicity of the nanocomposites synthesised through chemical and green routes, in addition to the identification of the best approach for the green synthesis of nanocomposites on the basis of biological and toxicological investigations. Additionally, different methods, including X-ray diffraction (XRD), scanning electron microscopy-energy-dispersive X-ray spectroscopy (SEM-EDX), and UV-vis absorption spectrophotometry (UV-vis), were used to classify the distinctive green and chemical nanocomposites produced.

## 2. Materials and methods

### 2.1. Chemical synthesis of CS/SiO_2_/TiO_2_/CeO_2_/Fe_3_O_4_ nanocomposites (CSTCF(C))

#### 2.1.1. Chitosan nanoparticle preparation (C(C))

Chitosan solution was prepared by dissolving purified chitosan with sonication in 1% (w/v) acetic acid solution until the solution was transparent.^34,35^ Sodium tripolyphosphate (STPP) was dissolved in deionized water at a concentration of 0.1% (w/v). Then, the STPP solution was poured dropwise into the chitosan solution under magnetic stirring at 1000 rpm using a stirring bar. Then, the mixture was stirred for an additional 15 min. The formation of chitosan nanoparticles started spontaneously *via* the STPP initiated ionic gelation mechanism.^34^ The nanoparticles were separated by centrifugation at 9000 rpm for 45 min. Then the supernatants were discarded. The nanoparticles were extensively rinsed with distilled water. After centrifugation the chitosan nanoparticles were dried at 47 °C in a hot air oven for further use or analysis.^36^

#### 2.1.2. CS/SiO_2_ (CS(C))

5 g of synthesised chitosan nanoparticles were dissolved in 100 ml of distilled water. Then, 5 g of silica gel was added, and the mixture was stirred at 45 °C for 24 h. The product was washed and dried in an oven at 120 °C for 10 h.^37^

#### 2.1.3. CS/SiO_2_/TiO_2_ (CST(C))

5 g of chitosan nanoparticles with silica gel nanocomposite powder were mixed with 100 ml of deionized water under stirring. The 5 grams of titanium dioxide were added. The resulting solution was centrifuged and solid CST(C) was separated and washed with water, then dried at 500 °C.^38^

#### 2.1.4. Synthesis of cerium oxide nanoparticles (CeO_2_NPs-C)

One mole of cerium nitrate was mixed with 100 ml of distilled water and 3 moles NaOH with 50 ml of distilled water. The above solutions were mixed together under stirring. The prepared solutions were centrifuged and dried in an oven at 250 °C. Finally, the cerium oxide nanoparticles were synthesised.

#### 2.1.5. CS/SiO_2_/TiO_2_/CeO_2_ (CSTC(C))

5 g of cerium oxide nanoparticle powder were added to the main mixture CST(C) and stirred for 5 h. The product was filtered, washed and dried in an oven at 130 °C for 10 h. The CSTC(C) nanocomposites were prepared *via* the chemical method.^39^

#### 2.1.6. Synthesis of iron oxide nanoparticles (Fe_3_O_4_NPs-C)

One molar aqueous ferric chloride solution (50 ml) was slowly added to 50 ml of one molar ferrous sulphate and stirred for 20 min. Then, 2 g of NaOH was slowly added under vigorous stirring, and was stirred for 24 h. The samples were centrifuged and washed several times and were dried at 100 °C for 8 h. Finally, iron oxide nanoparticles were synthesised.

#### 2.1.7. CS/SiO_2_/TiO_2_/CeO_2_/Fe_3_O_4_ nanocomposites (CSTCF(C))

**Fig. 1:**
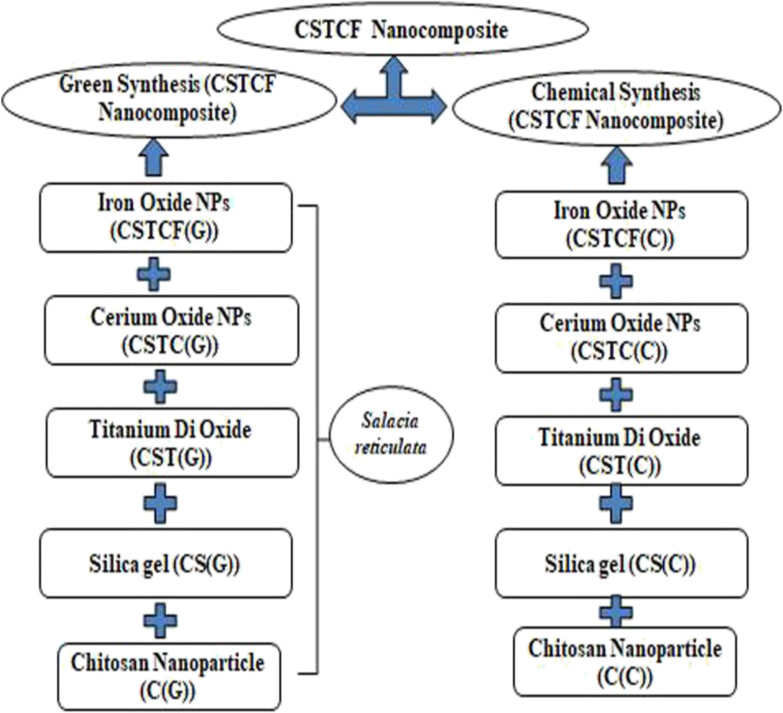
Schematic diagram of the green and chemical synthesis of CSTCF nanocomposites.

5 g of iron oxide nanoparticles were mixed with 100 ml of distilled water. Then, 5 g of dried CSTC(C) nanocomposite powder were added, and stirred for 24 h. The products were washed several times and dried at 100 °C for 8 h (Fig. 1).^40^

### 2.2. Green synthesis of CS/SiO_2_/TiO_2_/CeO_2_/Fe_3_O_4_ nanocomposites CSTCF(G)

#### 2.2.1. Preparation on *Salacia reticulata* leaf extract

10 g of leaf extract of dried *Salacia reticulata* was boiled in a solution of 70% water and 30% ethanol for 15 min. Then, the extract was filtered using filter paper, followed by centrifugation to achieve a liquid with no solid substances.

#### 2.2.2. Chitosan nanoparticle preparation C(G)

In a typical one-step synthesis protocol, 50 ml of 0.75% (w/v) chitosan solution was prepared using 0.1% acetic acid solution (in distilled water). Then, distilled water was added and the mixture was stirred using a magnetic stirrer at 70 °C for 12 h until the reaction was complete. In brief, the chitosan–STPP mixture was obtained by dissolving 0.8 g of sodium tripolyphosphate in 100 ml of chitosan solution. After 12 h the colloid was centrifuged at 10 000 rpm for 10 min to separate the particles from the suspension. Then, CSNPs were developed by adding 100 ml of the aqueous *Salacia reticulata* extract to 200 ml of a chitosan–STPP mixture dropwise under constant stirring for 30 min. The precipitate was re-suspended in acetone (90%, v/v) and the centrifugation was repeated three times to remove unreacted reagents. Finally, the precipitate was dissolved in water, dried in an oven overnight and stored.^41^

#### 2.2.3. CS/SiO_2_ nanocomposites (CS(G))

Chitosan nanoparticles were synthesized according to a green synthesis method, which used plant extract. In brief, a chitosan–silica doped mixture was obtained by dissolving 5 g of silica gel in 100 ml of chitosan solution, which was prepared by adding about 5 g of chitosan nanoparticles from the green synthesis method and then continuously magnetically stirring for 30 min. Then, the chitosan–silica gel was generated by adding 100 ml of the aqueous green *Salacia reticulata* extract to 100 ml of a chitosan–silica gel mixture dropwise under constant stirring for 30 min, then the mixture was heated at 60 °C for 4 h. After discarding the supernatant through centrifugation at 10 000 rpm for 20 min, the pellets were washed with distilled water and then dried in a hot air oven to obtain the nano doped particle powders.^42,43^

#### 2.2.4. CS/SiO_2_/TiO_2_ (CST(G))

For preparing the CST(G) mixture, 5 g of chitosan–silica green synthesised powder were dissolved in 100 ml of water, then stirred constantly with a magnetic stirrer and heated for 20 min at 90 °C. The mixture was mixed well, then sonicated for 10 min in a bath-type ultrasound sonicator to obtain a NP solution. Titanium dioxide (5 g) was then dissolved into 50 ml of the extract, and then the green chitosan–silica solution was added and the mixture was heated for 20 min at 90 °C under stirring. Finally, the solution was put through filtration, ultrasound, and drying.^44,45^

#### 2.2.5. Green synthesis of cerium oxide nanoparticles (CeO_2_NPs-G)

Cerium oxide nanoparticles were obtained by using cerium nitrate as a precursor. 1 molar concentration of cerium nitrate was made up to 50 ml. 50 ml of one molar NaOH was added to the cerium nitrate solution in a drop-by-drop manner under stirring. 50 ml of plant extract were added to the above solution. The final mixture solution was stirred for 5 h. Then the solution was filtered using filter paper, followed by centrifugation to achieve the solid with no liquid substances. Finally, the green synthesized cerium oxide nanoparticles (CeO_2_NPs-G) were prepared.

#### 2.2.6. CS/SiO_2_/TiO_2_/CeO_2_ nanocomposites (CSTC(G))

The CST(G) nanocomposite (5 g), which was obtained by the green synthesis method, was added to 100 ml of distilled water. Then, 5 g of cerium oxide nanoparticles (from the green synthesis) were added and stirred for 1 h. The obtained solutions were magnetically separated, washed repeatedly with ethanol and deionized water, and dried at 60 °C for 12 h.^46,47^

#### 2.2.7. Green synthesis of iron oxide nanoparticles (Fe_3_O_4_NPs-G)

Iron oxide nanoparticles were synthesized using ferric chloride and ferrous sulphate. 1 M ferric chloride in 50 ml of water and 1 M ferrous sulphate in 50 ml of water were mixed evenly, then 1 M NaOH in 50 ml of water was added. After 30 min, 50 ml of plant extract were added. The solution was heated at 200 °C for 12 h and the product was collected by a magnet and washed with deionized water and absolute ethanol. The product was dried in a hot air oven at 60 °C for 10 h.^48^

#### 2.2.8. Green CS/SiO_2_/TiO_2_/CeO_2_/Fe_3_O_4_ nanocomposites (CSTCF(G))

5 g of CSTC(G) (green synthesized nanocomposite) were added to 100 ml of distilled water and 5 g of iron oxide nanoparticles (green synthesized) were added. The solution was stirred using a magnetic stirrer at room temperature for 1 h. The solution was centrifuged at 10 000 rpm for 10 min, then the supernatant was discarded and the particles were collected (Fig. 1). The resulting dried sample was crushed into a powder and stored in an airtight container for further analysis.^49,50^

### 2.3. Photocatalytic activity

The photocatalytic performances of the CSTCF(G) nanocomposite and CSTCF(C) nanocomposite were evaluated by monitoring the photocatalytic degradation of malachite green dye under UV irradiation. In a typical procedure, 0.1 g was added to 100 ml of an aqueous solution of malachite green dye with an initial concentration of 1 ppm. Prior to irradiation, the suspension containing the CSTCF(G) nanocomposite, CSTCF(C) nanocomposite and dye solution was stirred in the dark for 30 min to achieve an adsorption/desorption equilibrium. Then, the suspension was irradiated with UV light. During the irradiation, about 2 ml of the suspension was taken from the mixture at regular intervals (30 min) and centrifuged to separate the photocatalyst particles. Then, the supernatant was analyzed by UV-vis spectrophotometry to measure the concentration of the malachite green dye solution, which exhibits a characteristic absorption at *λ*_max_ = 618 nm.^106,107^

The degradation efficiency was calculated using the formula below
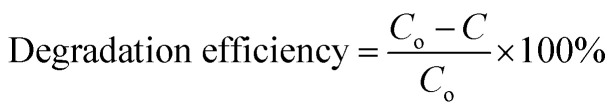
where *C*_o_ = initial malachite green dye concentration and *C* = concentration of the malachite green dye solution after the degradation time ‘*t*’.^106,107^

### 2.4. Antidiabetic activity

#### 2.4.1. α-Glucosidase inhibitory assays

Glucosidase inhibition assay of the green and chemically synthesised CSTCF(G) and CSTCF(C) nanocomposites was carried out as per Gosh *et al.*^89^ 100 μl of α-glucosidase (0.1 unit per ml) was combined with 200 μl of the CSTCF(G) and CSTCF(C) nanocomposites and incubated at 37 °C for 2 h. The enzyme was activated by adding 15 mM *p*-nitrophenyl d-glucopyranoside to a 100 mM phosphate buffer with a pH of 6.5, and stopped by adding 4 ml of 0.2 M Na_2_CO_3_ after 15 min at 37 °C. The absorbance of the *p*-nitrophenol produced by PNPG at 405 nm was measured using a 96-well plate reader to assess the α-glucosidase activity, considering the amount of enzyme required to generate one unit of α-glucosidase activity. Under test conditions, one unit of α-glucosidase activity is defined as the quantity of enzyme that hydrolyzed 2 M of *p*-nitrophenyl pyranoside in one minute.^51^*I*_α-glucosidase_ (%) = *A*_405control_ − *A*_405sample_/*A*_405control_ × 100

### 2.5 Anti-inflammatory activity

As a tough response to biological reactions from injured cells (local tissue injury), inflammation stops tissue damage and microbial infiltration through tiny wounds, scratches, and abrasions.^52^ The usual reagent for denaturing bovine serum albumin (BSA) is diclofenac sodium. In order to investigate the scavenging activity as previously mentioned, the BSA denaturation process was inhibited using both the green and chemically synthesized CSTCF nanocomposites.^53^ Dimethylformamide (DMF) was used to dissolve the chemically and green synthesized CSTCF nanocomposites, which were then diluted with phosphate buffer (0.2 M, pH 7.4). The resultant DMF concentration was maintained at 2.5% in each of the solutions. 1 ml of BSA (1 mM) was combined with 4 ml of the nanocomposites at a range of concentrations (10–50 μl ml^−1^), and the mixture was heated to 51 °C for 20 min. After the samples had been cooled to room temperature, a UV-vis spectrophotometer was used to calculate the turbidity to be 660 nm.^54^%*inhibition*=[{*A_control_*−*A_sample_*}/*A_control_*]×100

### 2.6. Cytotoxicity

#### 2.6.1. MTT (3-(4,5-dimethylthiazolyl-2)-2,5-diphenyltetrazolium bromide) assay

The MTT assay was used to assess the cytotoxicity of the green and chemically synthesised CSTCF(G) and CSTCF(C) nanocomposites on Pane1 cells. This method is based on the ability of viable cells to produce blue formazan crystals from yellow tetrazolium salt MTT *via* mitochondrial dehydrogenase. The obtained cells were placed in a plate with 96 cells at a density of 104 cells per well. Then, we chose cells with various nanocomposite concentrations (0.1, 0.5, 1, 5, 10, 50, and 100 μl ml^−1^) and incubated the microplate at 37 °C with 5% CO_2_ for one and two days. Then, we added 10 μl of MTT reagent to each well and incubated the plate for 4 h. Then, the supernatants were discarded, 100 μl of DMSO (dimethylsulfoxide) were added to each well, and the plates were incubated for 20 min in sequence. Finally, we were able to measure the cytotoxicity by measuring the absorbance at a suitable wavelength (*λ* = 570 nm) using an ELISA plate reader (Lab System). The percentages of cell cytotoxicity and viability were calculated using the following formula:^55^
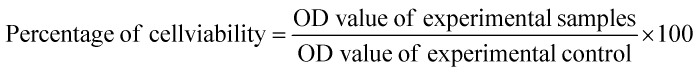


### 2.7. Antibacterial activity

#### 2.7.1. Test microorganism and microbial inoculum

Pathogenic microorganisms such as *E. coli*, *Bacillus* sp, *Enterobacter*, *Pseudomonas* sp, and *Staphylococcus aureus* were chosen for this study. Both bacterial strains were grown in nutritional agar medium at 37 °C (the microscopic organisms were grown in a nutrient broth at 37 °C and stored on nutrient agar inclines at 4 °C).

#### 2.7.2. Agar well diffusion method

The green and chemically synthesised CSTCF(G) and CSTCF(C) were tested for antibacterial activity against pathogenic germs such as *Escherichia coli*, *Bacillus* sp, *Enterobacter*, *Pseudomonas* sp and *Staphylococcus aureus*.^56^ The Muller Hinton agar containing the microbial inoculum was evenly distributed throughout the Petri plate. It was allowed to cool for a time before a well of 8–10 mm across was punched aseptically using a sterile cork borer or a tip. The well was filled with nanocomposites at concentrations of 20, 40, 60, and 80 μl ml^−1^, and the agar plates were cultured under sterile conditions with the test microorganism. The nanocomposites infiltrated into the agar media and inhibited organism growth. The presence of the restraining zone around the agar well at the time distinguished the antibacterial movement of the nanocomposites. The zone was calculated using a straight ruler from one edge of the reasonable region to the next.

### 2.8. Fish maintenance and breeding of zebrafish embryos

Zebrafish (*Danio rerio*) were housed in separate portions that were filled with the appropriate water (75 g of NaHCO_3_, 18 g of ocean salt, 8.4 g of CaSO_4_, per 1000 L). We housed wild-type grown-up zebrafish (Amphibian Environments) in a separate framework (Sea-going Living Spaces), and maintained and bred zebrafish as previously described.^57^ The night before production, two sets of developing male and female fish (proportion = 2 : 1) were placed in an incubation box, and a light (14 h)/dark (10 h) cycle was used to trigger variation and treatment of the undeveloped organisms. Production began the next morning when the light was turned on and was completed in one hour. Viable eggs were collected in a Petridish and flushed with E_3_ medium numerous times. E_3_ medium^58^ is standard incubator water for zebrafish eggs, and includes 5 mM NaCl, 0.17 mM KCl, 0.33 mM CaCl_2_, and 0.33 mM MgSO_4_, at pH 7.2–7.3, with broken down oxygen >6.3 mg L^−1^, full hardness 65 mg L^−1^ (as CaCO_3_), and a temperature of 28 ± 1 °C.^57^ Following international criteria for animal care, the institutional animal ethics committee of Sri Paramakalyani Centre for Environmental Science campus Manonmaniam Sundaranar University, Alwarkurichi, approved all the protocols.

#### 2.8.1. Embryo toxicity test for CSTCF(G) and CSTCF(C)

Schulte and Nagel^59^ developed the incipient organism test system, which is the foundation of this test. Hundreds of viable eggs were transferred into 96-well multi-plates, with 10 viable eggs in each well.^58^ Ten wells were filled with 2 ml each of green and chemically generated CSTCF(G) and CSTCF(C) nanocomposite and 2 ml of E_3_ medium as a control. All 96-well multi-plates were covered with clear plastic film and kept at 28 ± 1 °C with a 14 h/10 h light/dim photoperiod in an illuminated hatchery. The CSTCF(G) nanocomposite exposure experiments were carried out in the same way as the CSTCF(C) nanocomposite exposure experiments. The nanocomposites CSTCF(G) and CSTCF(C) were exposed at 24 hour intervals. A light magnifying tool and a camera device were used to observe the wells at predetermined time intervals (24, 48, 72 and 96 hpf). Mortality, gastrula improvement, tail separation, eyes, circulatory framework, heartbeat, pigmentation, and hatching length were among the toxicological endpoints. The chorion was totally removed from the unhatched eggs with young after 96 h of exposure. Every 24 h, the embryonic death and hatching rate were assessed. The embryo/larvae mortality and embryo hatching rate were employed as endpoints to investigate the developmental toxicity. Malformations were identified and photographed in both the control and treatment groups of the embryos and larvae. The hatching rate of the zebrafish was calculated using advanced image analysis (Scion Picture, ver. 4.0.3.2.). All the trials were repeated numerous times independently, and the percentage of defective embryos was measured every 24 hours.

### 2.9. Statistical analysis

GraphPad Prism software (version 8.3.4 for Windows) was used to evaluate the data statistically using one-way analysis of variance (ANOVA) and Dunnett's multiple range test (Tukey's *post hoc* test) (GraphPad Software, La Jolla, California, USA). The data are presented as mean ± standard deviation (SD) from three independent experiments for all the experiments.

## 3. Results and discussion

### 3.1. X-ray diffraction

Variation in the broadness of the peaks was observed in the XRD spectra from the green and chemical methods.

**Fig. 2:**
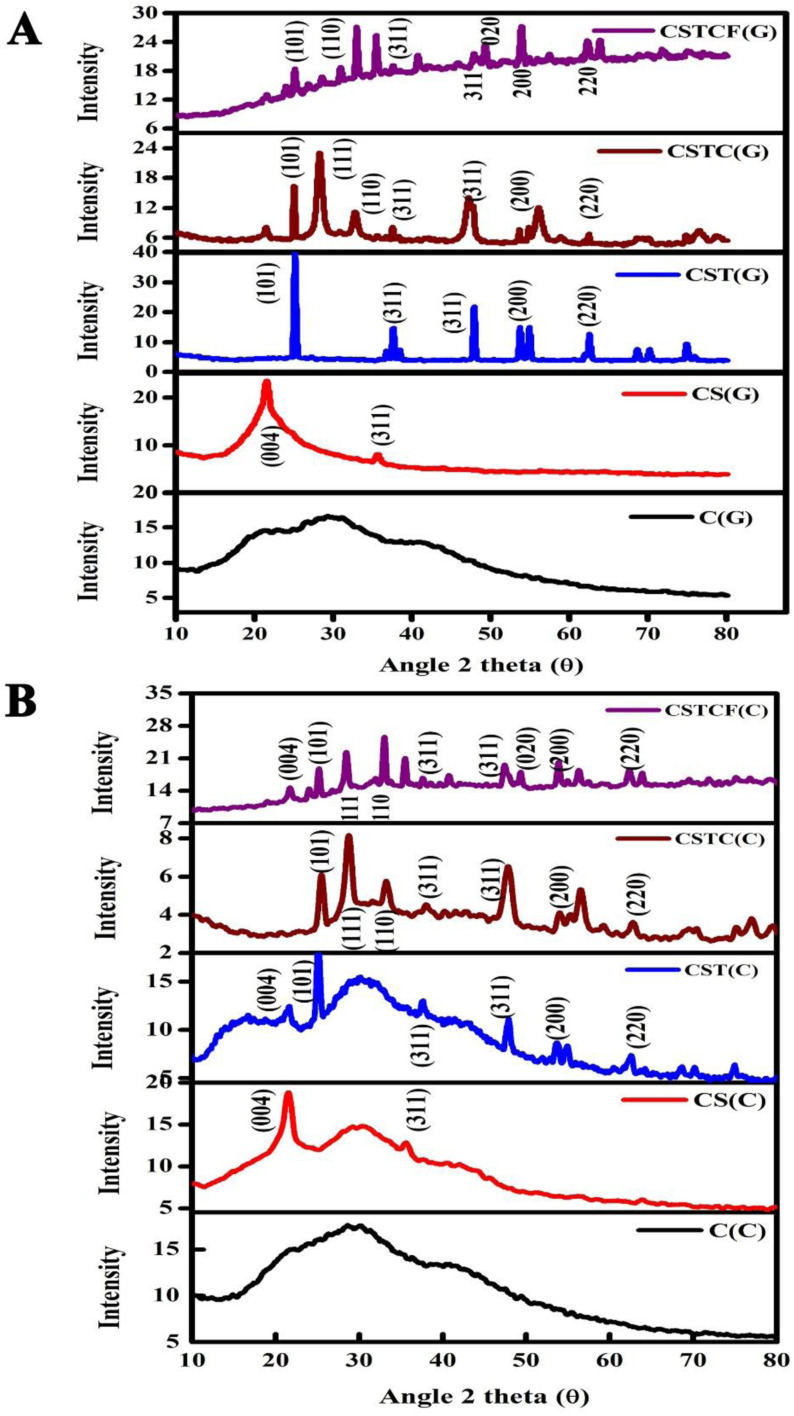
XRD images of the CSTCF nanocomposite. (A) Shows the green synthesized CSTCF(G) nanocomposite and (B) shows the chemically synthesized CSTCF(C) nanocomposite.

The green and chemically synthesized nanoparticles and nanocomposites prepared at room temperature with low crystallinity are shown as C, CS, CST, CSTC, and CSTCF in Fig. 2. Fig. 2(A) shows the green synthesized CSTCF(G) nanocomposite, and Fig. 2(B) shows the chemically synthesized CSTCF(C) nanocomposite. The figures show that there is a series of diffraction peaks that are the same across the samples – for the green synthesized CSTCF(G) nanocomposite, these are observed at (101), (110), (311), (020), (311), (200), and for CSTCF(C) these are observed at (004), (101), (111), (110), (311), (311), (020), (200), (220). No diffraction peaks matching the chitosan nanoparticles and SiO_2_ can be observed, indicating that chitosan nanoparticles and SiO_2_ are in an amorphous phase. TiO_2_ is represented in both the samples, which indicate a series of diffraction peaks at the positions 25.32°, 27.52°, and 48.00°, with planes such as (101), (004), and (200), which are compatible with the pure phase of tetragonal TiO_2_. CeO NPs are suggested by a series of diffraction peaks observed at 24.03°, 32.04° and 47.86°, corresponding to (200), (112) and (312). Good consistency between the pure phases of tetragonal CeO_2_ NPs are observed. Additionally, the addition of nanoparticles like CeO_2_ NPs and Fe_3_O_4_ NPs to the nanocomposite layer might cause a decrease in the intensity of the three preceding components, resulting in an increase in the crystal size of the nanocomposites.^60,61,104^ As a consequence, the particle sizes of the CSTCF(G) nanocomposite and CSTCF(C) nanocomposite were discovered to be equivalent, with the CSTCF(G) nanocomposite having a smaller size. The results showed that the CSTCF(G) nanocomposite exhibited a decrease in particle size and an increase in surface area. Fig. 2(A) shows unmistakably how different methods, plants, and extraction techniques affect the strength and quality of the XRD peaks. The XRD patterns showed well-defined reflection peaks, indicating that the produced nanoparticles and nanocomposites had a high degree of crystallinity. The shape of nanoparticles varied due to differences in the synthesis method. Heat was used to prepare the *Salacia reticulata* extract, which may have degraded the biomolecules responsible for the conversion of ions into nanocomposites. As a result, the nanocomposite generated by the *Salacia reticulata* extract was crystalline. The size of the particles can be calculated using the Debye–Scherrer equation^62,94–97,102^*D* = 0.92*λ*/*β* cos *θ*where *D* is the particle size, *K* is a shape factor (it is a constant approximately equal to 0.9), *λ* is the wavelength of the X-rays (wavelength of Cu Kα radiation, 1.5418 Å), *β* is the full line width at half-maximum (FWHM) of the main intensity peak, and *θ* is the Bragg angle.^105^ The broad diffraction peaks observed in the case of the green synthesized nanocomposite confirms the smaller size of the particles compared to the chemically synthesized nanocomposites. The average particle size of the chemically synthesized CSTCF(C) nanocomposites is 36 nm, and that for the green synthesized CSTCF(G) nanocomposites is approximately 27 nm.

### 3.2. Fourier-transform infrared (FTIR) spectroscopy

**Fig. 3:**
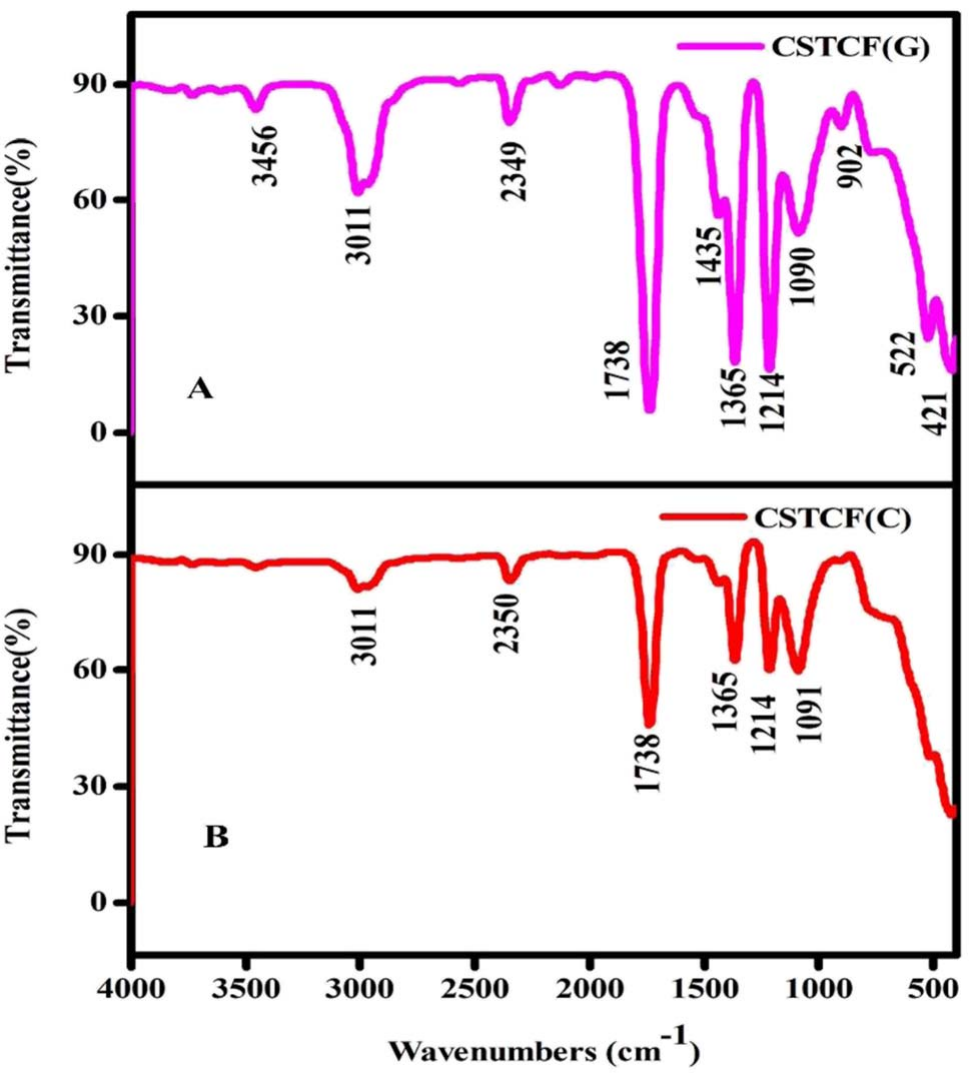
FTIR image of the CSTCF nanocomposites. (A) Shows the green synthesized CSTCF(G) nanocomposite and (B) shows the chemically synthesized CSTCF(C) nanocomposite.

The FTIR spectra of the green and chemically manufactured CSTCF nanocomposite powders were obtained between 500 and 4000 cm^−1^ and are displayed in Fig. 3.

The presence of functional groups in the produced samples was determined using symmetric and asymmetric stretching. The CSTCF(G) nanocomposite showed characteristic peaks at 3456 cm^−1^, 3011 cm^−1^, 2349 cm^−1^, 1738 cm^−1^, 1435 cm^−1^, 1365 cm^−1^, 1214 cm^−1^, 1090 cm^−1^, 902 cm^−1^, 522 cm^−1^, and 421 cm^−1^. Characteristic peaks for the CSTCF(C) nanocomposite were shown at 3011 cm^−1^, 2350 cm^−1^, 1738 cm^−1^, 1365 cm^−1^, 1214 cm^−1^, and 1090 cm^−1^. It is likely that the peak of the green synthesized nanocomposite at 1435 cm^−1^ represents acyl C–O (or phenol C–O) stretching or sp^3^ C–H bending, whereas that at 902 cm^−1^ denotes C–H stretching. Furthermore, an alkoxy C–O peak at 522 cm^−1^ is indicated. Due to the potential existence of bioactive phytochemicals, unsaturated C–H bending emerges below 421 cm^−1^, and the weak S–S stretching vibration occurs between 900 and 400 cm^−1^. For CSTCF(C), the presence of water is indicated by the occurrence of the bending mode at around 1738 cm^−1^ and the stretching mode at around ∼3011 cm^−1^ in all the spectra.^55,63^ A prominent peak of Fe_3_O_4_ was found at 522 cm^−1^, which was assigned to the Fe–O stretching vibration. In Fig. 3, there was a new strong band at about 1214 cm^−1^ that came from the Si–O bond in SiO_2_.^63^ There was also the vibration band for the Ti–O–Ti bond fingerprint, which is positioned around 1091 cm^−1^.^55,63^ The appearance of a prominent peak at 902 cm^−1^ is caused by the Ce–O stretching vibration of CeO_2_.^64^ Plant extracts with a higher concentration of phenolics and flavonoids, which are responsible for their antioxidative response, may have a role in the reduction, capping, and stabilisation of the NP production. As a result, several functional groups are discovered on the nanoparticles and generated by the plant extract. Variation in *Salacia reticulata* plant extracts is attributed to diverse phytocomponents. The FTIR spectra also demonstrate that the green synthesis processes results in the attachment of functional groups to the surfaces of the nanoparticles and nanocomposites, with the intensity of each peak varying depending on the extract utilised.^65^

### 3.3. Ultra violet spectroscopy

**Fig. 4:**
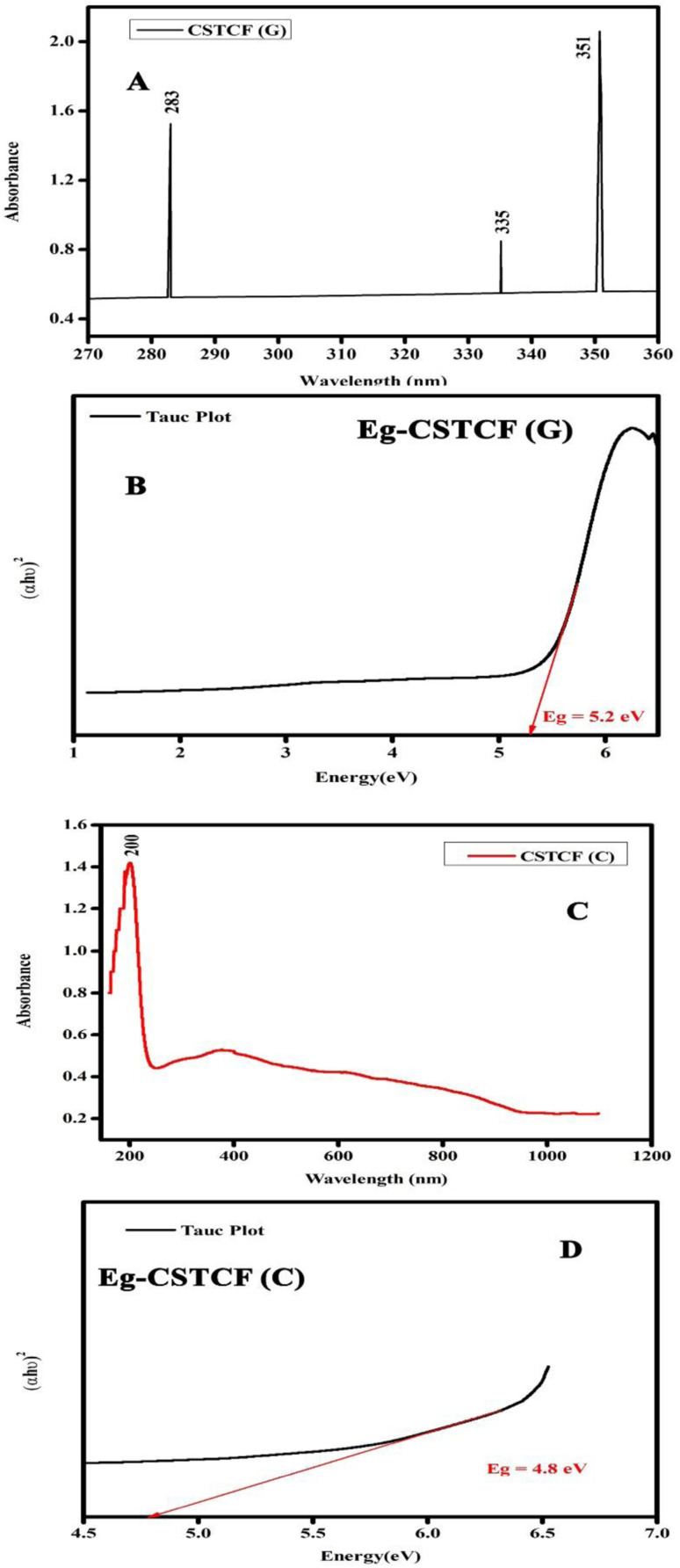
UV image of the CSTCF nanocomposites. (A) Shows the green synthesized CSTCF(G) nanocomposite. (B) Shows the band gap of the green synthesized CSTCF(G) nanocomposite. (C) Shows the chemically synthesized CSTCF(C) nanocomposite. (D) Shows the band gap of the chemically synthesized CSTCF(C) nanocomposite.

In the wavelength range of 200–900 nm, the optical absorption coefficient was computed. UV-vis measurements were taken to define a suspension where reliable band gap energy measurements could be taken.^66^ The UV-vis spectra of the green and chemically produced nanocomposite are shown in Fig. 4. The spectra demonstrate that the green synthesised nanocomposite (Fig. 4(A)) has absorbance peaks in the UV area at wavelengths of 283 nm, 335 nm, and 351 nm, whereas the chemically generated nanocomposite (Fig. 4(C)) has an absorbance peak at a wavelength of 200 nm. Similar to the spectrum described previously, the wavelength of 283 nm represents the presence of iron oxide nanoparticles, 335 nm represents the presence of titanium dioxide, and 351 nm represents the presence of cerium oxide nanoparticles.^67^ Finally, the UV spectrum in Fig. 4(A) reveals the formation of three distinct peaks at three different wavelengths, indicating a physical mixture of a few nanoparticles. Fig. 4(C) shows a UV spectrum with a single hump, there are also vibrational and rotational energies present. The highest absorbance is obtained at shorter wavelengths as the particle size decreases, resulting in a blueshift as the wavelength decreases.

A broad size distribution leads to a broad absorption maximum, and the reverse is also true.^68,69^ We can describe the discrepancy in the quantum size effect, which states that the size of the particles affects their band gap energy. Because nanomaterials' energy band gaps are inversely related to their diameters, it's straightforward to claim that regulating their sizes can influence their energy band gaps.^70^ The band gap of the nanoparticles as synthesised is calculated using the equation:(*αhγ*)^2^ = *C*(*hγ* − *E*_g_)where *C* is a constant, *E*_g_ is the material's band gap, and *α* is the absorption co-efficient. The energy bandgap value of the CSTCF(C) nanocomposites is 4.8 eV, and that of the CSTCF(G) nanocomposites is 5.2 eV.

### 3.4. Scanning electron microscopy-EDX

**Fig. 5:**
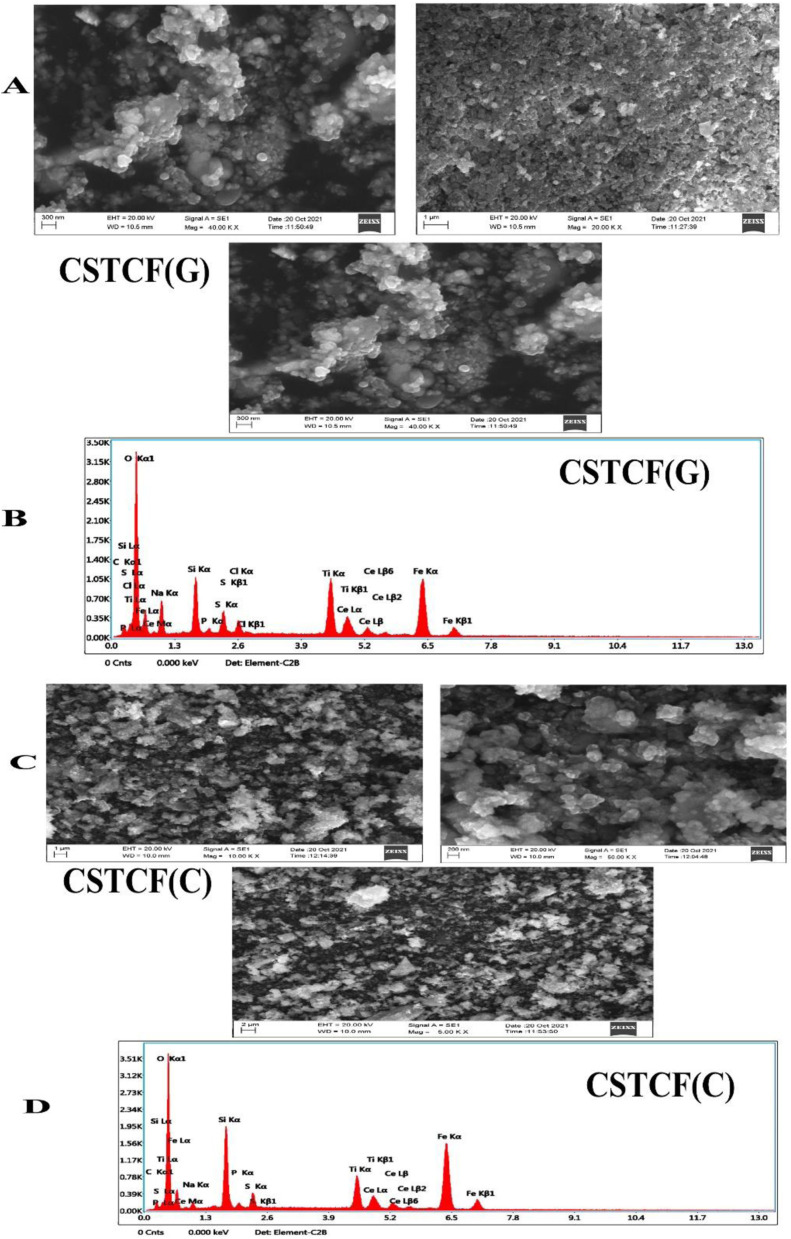
SEM-EDX images of the CSTCF nanocomposites. (A) Shows the SEM image of the green synthesized CSTCF(G) nanocomposite. (B) Shows the EDX image of the green synthesized CSTCF(G) nanocomposite. (C) Shows the SEM image of the chemically synthesized CSTCF(C) nanocomposite. (D) Shows the EDX image of the chemically synthesized CSTCF(C) nanocomposite.

Fig. 5(A)–(D) show the SEM results of the CSTCF(C) and CSTCF(G) nanocomposite under low and high magnification. Both have the same shape (spherical) and a homogeneous distribution morphologically, with some particles clumping together. The surface of the iron oxide nanoparticle coated nanocomposite had surface roughness, which allowed the organic component to make good contact with the catalysts.^71^ The nanocomposite CSTCF(G), with diameters of 10–30 nm, and CSTCF(C), with diameters of 10–40 nm, were found to be successfully synthesised. An SEM image of a green and chemically manufactured nanocomposite was also captured, with the darker parts corresponding to the magnetic component and the lighter portions corresponding to the remaining phases that make up this nanostructure. Furthermore, the EDX intensities of Ti, Fe, Si, Ce, and O peak in the produced samples as predicted for the green and chemically manufactured nanocomposite in Fig. 5(B) and (D).

### 3.5. Mechanism of photocatalytic degradation of the dye

**Scheme 1:**
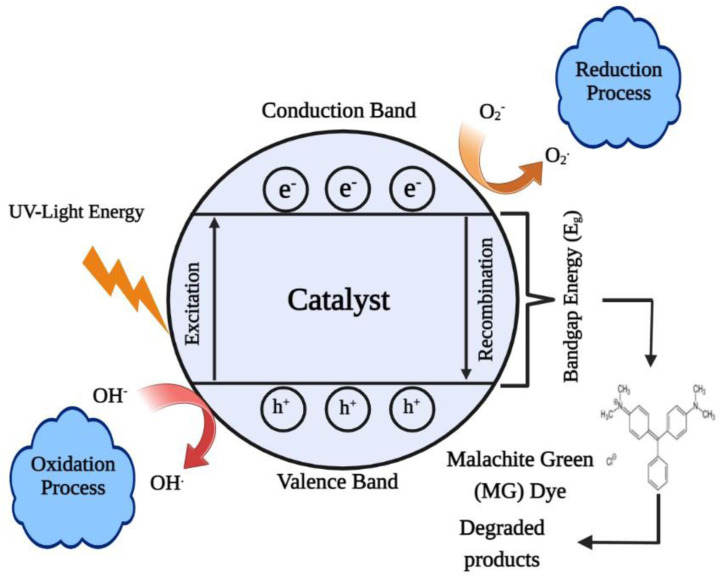
Reaction mechanisms for the degradation of malachite green.

The electronic structure of the catalyst is critical in photocatalysis. The band gap is the energy difference between the valence band (VB) and conduction band (CB) levels in a catalyst. Without excitation, both electrons and holes are in the valence band. When the catalyst surface is exposed to light, electrons are transported from the VB to the CB by absorbing specific wavelengths, leaving holes (h^+^) in the valence band and thereby forming electron–hole pairs. Electrons and holes migrate to the catalyst surface and can reduce and oxidise the reactants that have been adsorbed by the catalyst, respectively. These photo-induced electrons and holes have substantially larger reduction and oxidation potentials than hydrogen and ozone. As a result, these electron–hole pairs form a powerful redox system. By oxidising OH and H_2_O molecules that are adsorbed on the catalyst surfaces, photo-produced holes generate hydroxyl radicals. Concurrently, electrons in the conduction band could aid in the reduction of O_2_ molecules in air adsorbed on the catalyst surfaces, eventually forming peroxyl radicals. These photo-generated hydroxyl and peroxyl radicals oxidise and destroy organic and inorganic materials. The reduction and oxidation reactions are the primary mechanisms of photocatalytic generation. Scheme 1 shows a schematic illustration of this.^108^ Within a relatively short time period, these photo-generated electrons and holes can recombine in the bulk or on the surface of the catalyst, releasing energy in the form of heat or photons.

#### 3.5.1. Photocatalytic activity

**Fig. 6:**
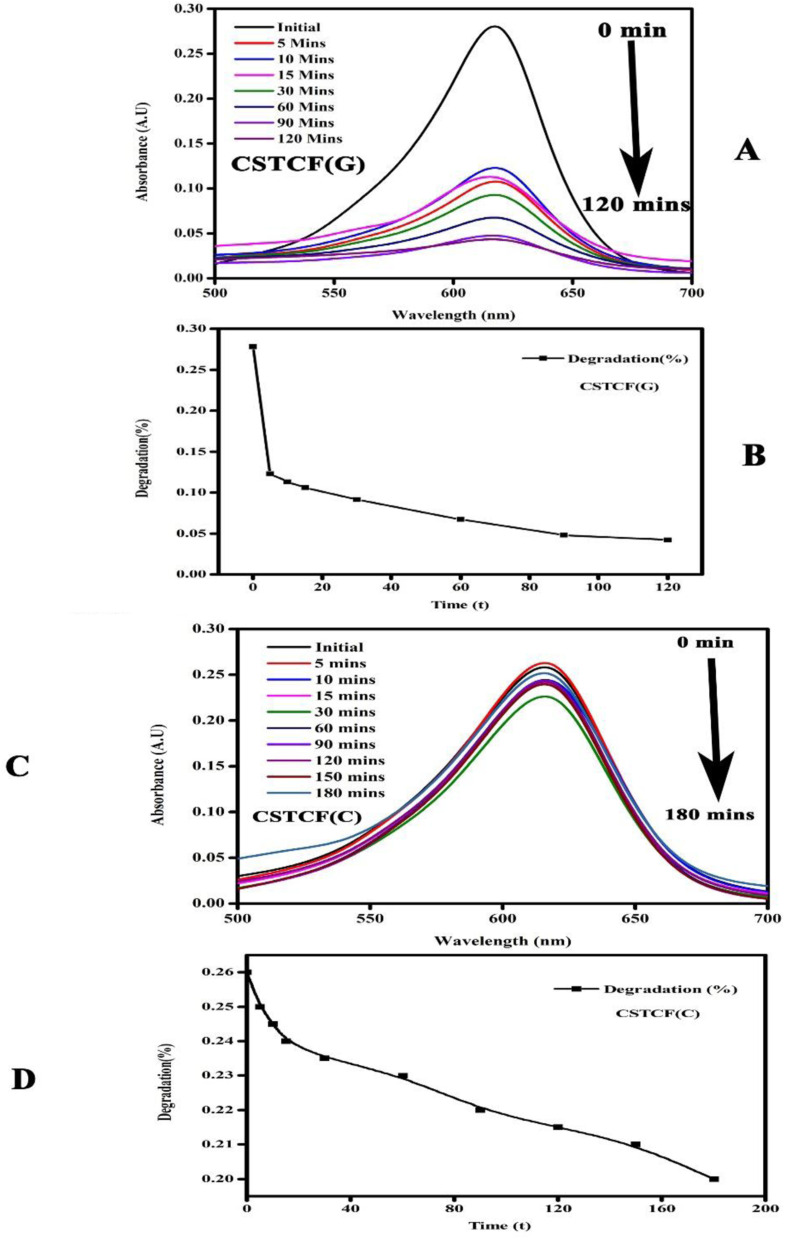
Photocatalysis images of the CSTCF nanocomposites. (A) Shows the green synthesized CSTCF(G) nanocomposite. (B) Shows the degradation of the dye using the green synthesized CSTCF(G) nanocomposite. (C) Shows the chemically synthesized CSTCF(C) nanocomposite. (D) Shows the degradation of the dye using the chemically synthesized CSTCF(C) nanocomposite.

The photocatalytic effect of the CSTCF(G) nanocomposite and CSTCF(C) nanocomposite photocatalysts under UV light is shown in Fig. 6(A–D).

It was observed that the CSTCF(G) nanocomposite is more effective than the CSTCF(C) nanocomposite samples under UV light, while in the presence of UV light the degradation percentage increases. Green synthesized photocatalysts show high photocatalytic activity under UV light as compared to the chemically synthesised CSTCF(C) nanocomposite for the degradation of MG. A very small amount of dye was degraded under UV light, which may be because of the rigid structure of the malachite green. The photocatalysis results shown in Fig. 6(A–C) specify that 2 to 3% MG was degraded after 180 min under UV light which is in accordance with reported values. According to previous research, the degradation of cationic dyes takes longer under visible light because visible light wavelengths have low energy.^98–100^ However, in this work, for the first time, it was discovered that the CSTCF(G) nanocomposite and CSTCF(C) nanocomposite have greater photocatalyst efficiency than those found in other previous investigations of magnetic photocatalysts under visible light.^98–100,106,107^

### 3.6. Anti-diabetic activity

#### 3.6.1. α-Glucosidase activity

**Fig. 7:**
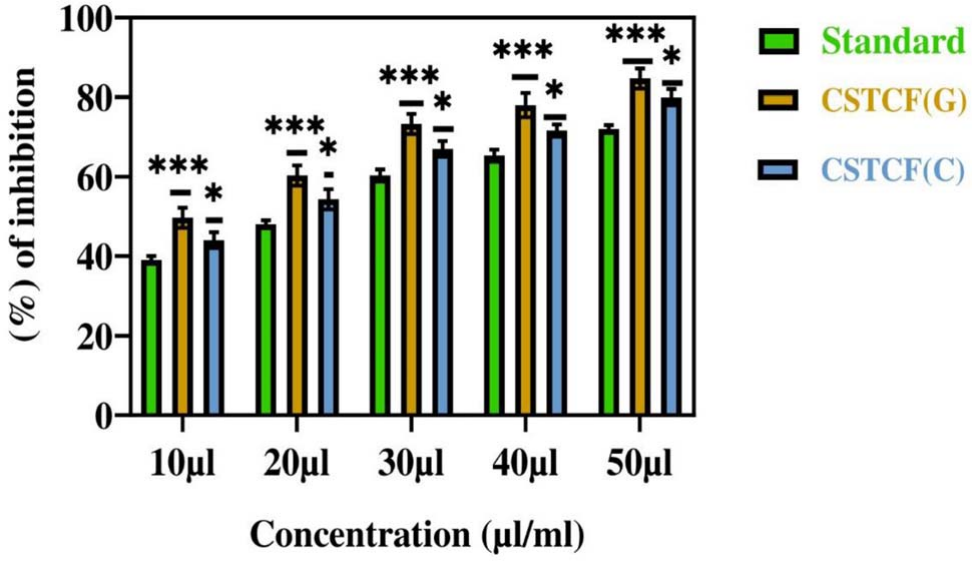
Anti-diabetic activity of the CSTCF(G) and CSTCF(C) nanocomposites. The data are presented as mean ± SD of three replications. The data were analysed statistically by one-way analysis of variance followed by Dunnett's multiple range test (Tukey's *post hoc* test) using GraphPad Prism software. Statistical significance: “*” and “***” represent *p* < 0.05 and *p* < 0.005.

The inhibition of digestive enzymes, such as α-glucosidase, considerably reduces higher blood glucose levels in diabetic conditions,^72,73^ hence the CSTSF(G) and CSTSF(C) nanocomposites were evaluated for *in vitro* α-glucosidase enzyme inhibitory activity (Fig. 7). The CSTCF(G) and CSTCF(C) nanocomposites exhibited percentage α-glucosidase activities of 79.29 ± 0.48 and 27.01 ± 5.01, respectively. The IC_50_ values of the antidiabetic activity for the green and chemically synthesized CSTSF nanocomposites were 26 μl ml^−1^ and 71 μl ml^−1^, respectively (Table 1). Accordingly, the green synthesized the CSTCF(G) nanocomposite significantly inhibits the α-glucosidase less than the CSTCF(C) nanocomposite.^74^

IC_50_ values of antidiabetic activity, anti-inflammatory activity, and cytotoxicity

**Table:** 

	Parameters	IC_50_ value (μl ml^−1^) – standard	IC_50_ value (μl ml^−1^) CSTCF(C)	IC_50_ value (μl ml^−1^) CSTCF(G)
Anti-diabetic activity	α-Glucosidase	25	71	26
Anti-inflammatory activity	BSA	64.3	87.7	50.7

**Table:** 

	Parameters	IC_50_ value (μl ml^−1^) – *Salacia reticulata*	IC_50_ value (μl ml^−1^) CSTCF(C)	IC_50_ value (μl ml^−1^) CSTCF(G)
Cytotoxicity	(HaCaT)	1.26	14.6	0.08
	(MCF-7)	0.9	2.8	0.05

### 3.7. Anti-inflammatory activity

The bovine serum albumin denaturation assay was used to gauge the anti-inflammatory effect of the CSTCF nanocomposites (green *vs.* chemical). For the anti-inflammatory experiments, different quantities (20–100 μl ml^−1^) of both CSTCF nanocomposites were used.

**Fig. 8:**
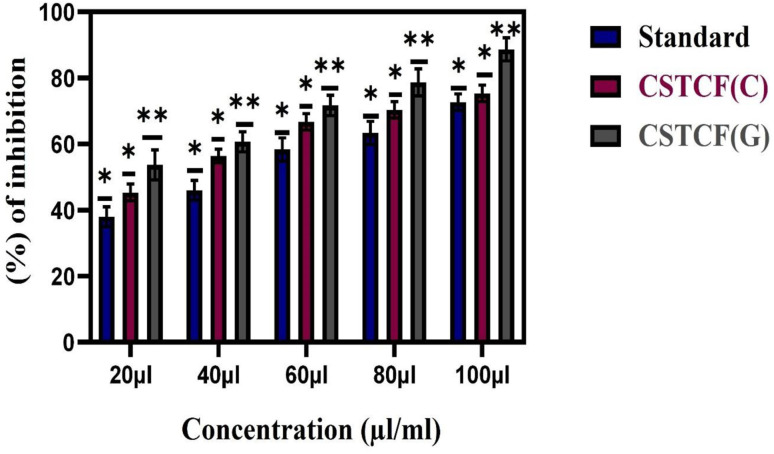
Anti-inflammatory activity of CSTCF(G) and CSTCF(C) nanocomposites. The data are presented as mean ± SD of three replications. The data were analysed statistically by one-way analysis of variance followed by Dunnett's multiple range test (Tukey's *post hoc* test) using GraphPad Prism software. Statistical significance: “*” and “**” represent *p* < 0.05 and *p* < 0.01.

Similar findings were made in a previous report,^75^ in which the BSA denaturation method was used to examine the anti-inflammatory activity of silver nanoparticles and nanocomposites.^75^ According to Fig. 8, the combined green mediated CSTCF(G) nanocomposites retained good anti-inflammatory properties and showed stronger anti-inflammatory properties than the standard solution and chemical mediated CSTCF nanocomposite. According to the anti-inflammatory results, shown in Table 1, inflammation is significantly reduced as compared to the standard solution (diclofenac sodium). The IC_50_ values for the chemical and green CSTCF nanocomposite anti-inflammatory activity were 87.7 μl ml^−1^ and 50.7 μl ml^−1^, respectively (Table 1). These findings showed that the green produced CSTCF nanocomposite is a superior agent for producing potent anti-inflammatory drugs. Similar to in previous work, these nanocomposites' effective anti-inflammatory capabilities may enable their use in biomedical applications and food packaging to prevent oxidative stress.^76^

### 3.8. Cytotoxicity

#### 3.8.1. MTT assay

**Fig. 9:**
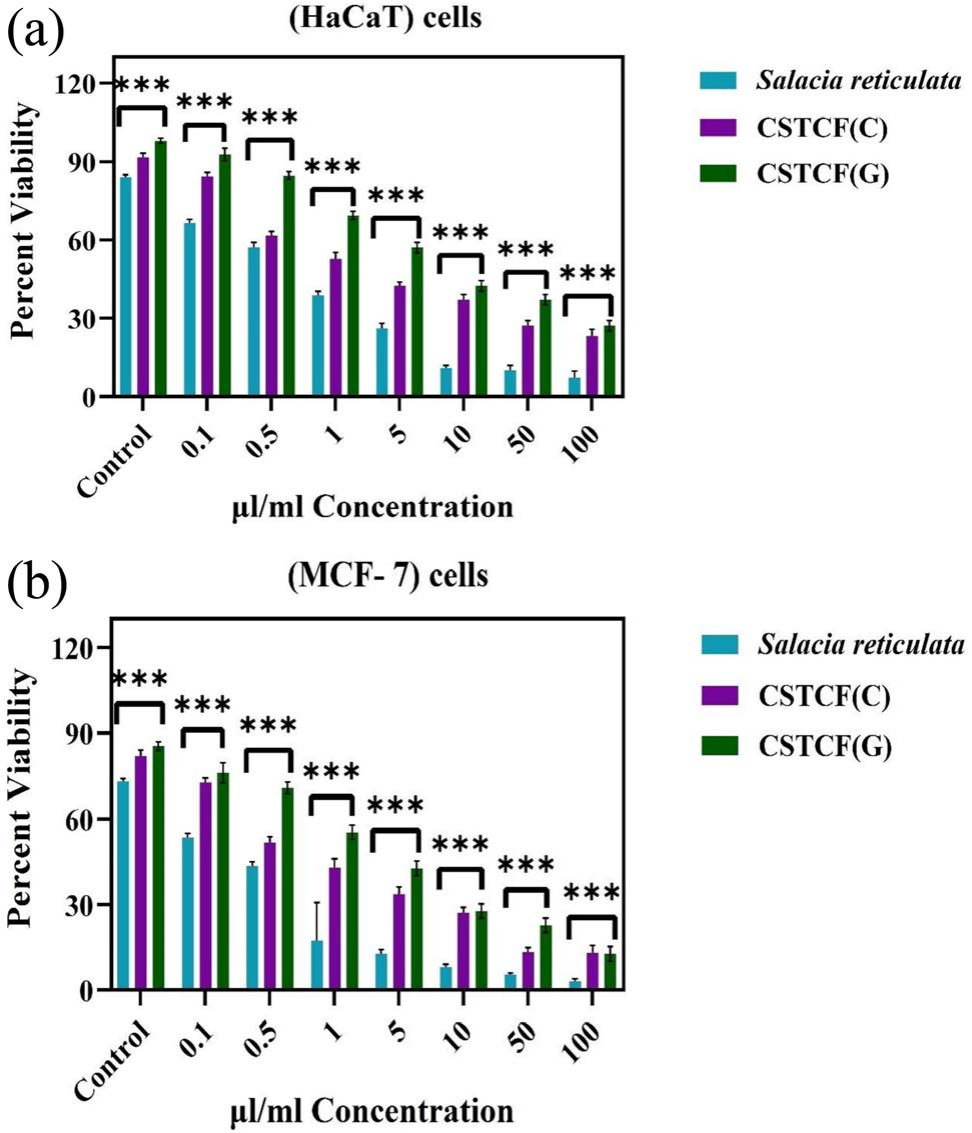
(a) *In vitro* cytotoxicity (cell viability) assay on normal human keratinocyte (HaCaT) cells after treatment with several concentrations of CSTCF(G), CSTCF(C), and plant extract (*Salacia reticulata*). Data represent mean ± standard deviations of three replications. The data were analysed statistically by one way analysis of variance followed by Dunnett's multiple range test (Tukey's *post hoc* test) using Graph Pad Prism Software. Bars labeled with “***” represent statistically significant results (*p* < 0.001). (b). *In vitro* cytotoxicity (cell viability) assay on human breast cancer (MCF-7) cells after treatment with several concentrations of CSTCF(G), CSTCF(C), and plant extract (*Salacia reticulata*). Data represent mean ± standard deviations of three replications. The data were analysed statistically by one way analysis of variance followed by Dunnett's multiple range test (Tukey's *post hoc* test) using Graph Pad Prism Software. Bars labeled with “***” represent statistically significant results (*p* < 0.001).

The MTT assay was used to confirm the nanoparticles' *in vitro* biocompatibility at various concentrations. Fig. 9(a and b) show the results that were attained. As can be observed, all of the samples were non-toxic at concentrations between 0.1 and 100 μl ml^−1^. In this work, the cytotoxicity of *Salacia reticulata* leaf extract and the CSTCF nanocomposite (green *vs.* chemical) was examined in human keratinocyte (HaCaT) and human breast cancer (MCF-7) cells (Fig. 9(a and b)). The cells were exposed to various concentrations of leaf extract and CSTCF nanocomposite (0.1–100 μl ml^−1^) for 24 h. The leaf extract and both forms of CSTCF nanocomposite decreased the viability of the two cells in a dose-dependent manner. The IC_50_ values for both types of CSTCF nanocomposite (green and chemical) for the HaCaT and MCF-7 cells are shown in Table 1. These results demonstrated that the green-produced CSTCF nanocomposite is more harmful to MCF-7 cancer cells than the chemically-produced CSTCF nanocomposite. The results of the current investigation are in agreement with several past studies. The amount of released CSTCF nanocomposite at the lower concentrations was sufficient to have a meaningful impact on the cell survival.

In a prior work, CuO nanoparticles and nanocomposite induced concentration-dependent cytotoxicity in human liver cancer (HepG_2_) cells.^77^ A previous report^74^ showed a similar trend of increased CoNPs and nanocomposite cytotoxicity against human cell line U937 by increasing the particle concentration.^74^ A concentration-dependent biocompatibility of Co NPs against HIF-1a and HIF-1a (2/2) cells was also found in earlier studies.^73^Fig. 8 illustrates how this work's effects on cell survival can be seen. While chemically synthesised CSTCF nanocomposite particles were harmful, green synthesised CSTCF nanocomposite particles showed less toxicity than CSTCF(C). Consideration of the lowered release of the CSTCF(G) nanocomposite caused by the presence of *Salacia reticulata* may help to explain this decreased toxicity of the CSTCF(G) nanocomposite. However, a difference in cell viability caused by increasing the density of the CSTCF(G) nanocomposite particles shows that the toxicity of these particles varies. Other studies that demonstrate that cell viability is reduced by increasing the CSTCF(G) nanocomposite concentrations have also characterised the dose- and concentration-dependent cytotoxicity of the CSTCF(G) nanocomposite against a variety of cell lines.

### 3.9. Zebrafish embryo toxicity

**Fig. 10:**
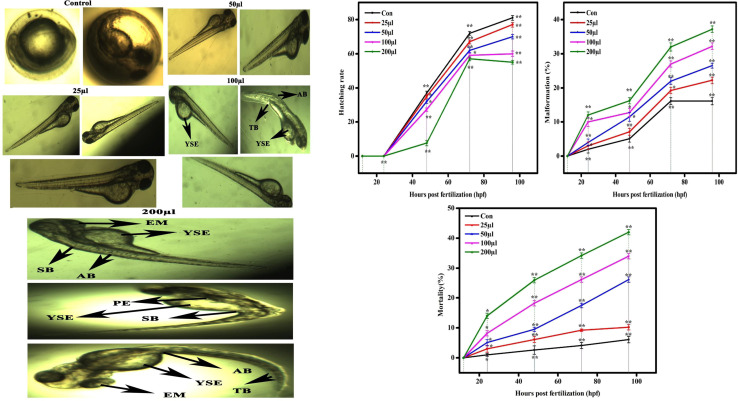
(a) Representative image of zebrafish embryos and larva exposed to CSTCF(G). The control group shows the normal appearance at the relevant concentration and number of hours. Tail bent (TB), yolk sac edema (YSE), eye malformation (EM), spinal curvature bent (SB), axis bent (AB) and pericardial edema (PE) are observed as malformations with the CSTCF(G) after exposure to 100–200 μl CSTCF(G) at 96 hpf. (b)Data for CSTCF(G) presented as mean ± standard deviation. The data were analysed statistically by one way analysis of variance followed by Dunnett's multiple range test (Tukey's *post hoc* test) using Graph Pad Prism software. Significance: “*” and “**” represent *p* < 0.05 and *p* < 0.01, respectively.

**Fig. 11:**
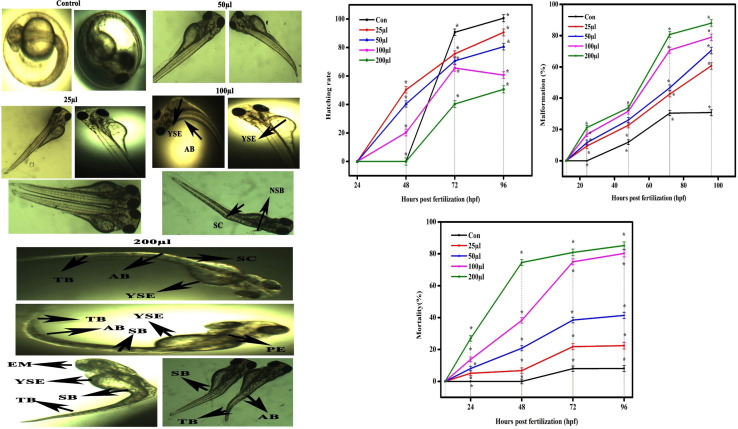
(a) Representative image of zebrafish embryos and larva exposed to CSTCF(C). The control group shows the normal appearance at the relevant concentration and number of hours. Tail bent (TB), yolk sac edema (YSE), eye malformation (EM), spinal curvature bent (SB), axis bent (AB), non-inflated swim bladder (NSB) and pericardial edema (PE) are observed as malformations with the CSTCF(C) after exposure to 100–200 μl CSTCF(C) at 96 hpf. (b) Data for CSTCF(C) presented as mean ± standard deviation. The data were analysed statistically by one way analysis of variance followed by Dunnett's multiple range test (Tukey's *post hoc* test) using Graph Pad Prism software. Significance: “*” represents *p* < 0.05.

The CSTCF nanocomposite (green *vs.* chemical) synthesised utilising both procedures showed differences in the hatching and mortality rates as well as developmental problems. Fig. 10(a, b) and 11(a, b) demonstrate the CSTCF nanocomposite's comparative toxicity (green *vs.* chemical). On the surface of the eggs, there was a significant deposit of green and chemically synthesized CSTCF nanocomposite. When compared to the control, the differences in the hatching rate of zebrafish embryos exposed to 25, 50, 100, and 200 μl ml^−1^ concentrations of the chemically synthesized CSTCF(C) nanocomposite were found to be statistically very significant (*p* ≤ 0.05). The differences in the rates for 25, 50, 100, and 200 μl ml^−1^ concentrations of the environmentally synthesized CSTCF(G) nanocomposite were also found to be statistically very significant (*p* ≤ 0.01), compared with the control. The hatching and death rates showed that the CSTCF nanocomposite's toxicity (green *vs.* chemical) was dose-dependent, and that the green technique was more toxic than the chemical method. Apart from the decreased hatching rate for 24–96 hpf, several zebrafish embryos that were experimented on with 100 μl ml^−1^ and 200 μl ml^−1^ concentrations of both the CSTCF nanocomposites (green *vs.* chemical) exhibited malformations (Fig. 10 and 11).

These malformations included spinal curvature bent (SB), pericardial oedema (PE), bent tail (TB), axis bent (AB), eye malformation (EM), non-inflated swimbladder (NSB), and yolk sac oedema (YSE). Table 2 also compares embryonic touch reactions following exposure to the CSTCF nanocomposite (green *vs.* chemical). The concentration had no effect on the frequency of these abnormalities in the embryo population. Hu *et al.*^78^ observed that exposure to a nanocomposite affected the mortality rate and hatching rate of zebrafish embryos in dose- and time-dependent ways.^78^ In zebrafish embryos, the toxicities of many other nanomaterials, such as nanocomposite nanoparticles, including ZnO NPs,^58^ TiO_2_ NPs,^79^ copper NPs,^80^ CuO NPs,^81^ ZrO NPs,^82^ SiO_2_ NPs^83^ and carbon nanotubes,^84^ and magnetite based nanocomposites,^85^ have been examined. Significant results include higher mortality, a lower hatching rate, and developmental abnormalities.^80–84^

**Table:** Comparison of touch swim responses of the CSTCF nanocomposite (green *vs.* chemical)

Concentration μl ml^−1^	Touch and swim response		Legend
	CSTCF(G)	CSTCF(C)	
Control	++++	++++	++++ Fast response
25	++++	+++	+++ Medium response
50	++++	++	++ Slow response
100	++++	+	+ Very Slow response
200	+++	—	− No response

### 3.10. Antibacterial activity

The antibacterial potential of the prepared nanocomposite was evaluated against three Gram-negative and two Gram-positive pathogenic strains *via* the agar well diffusion method.

**Fig. 12:**
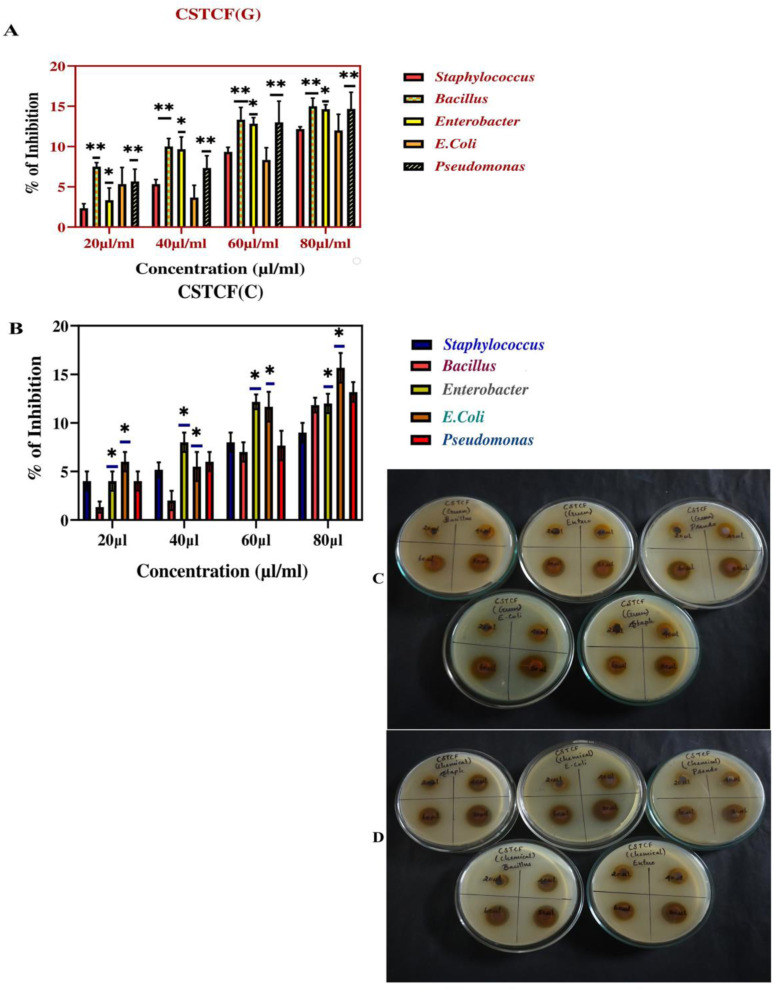
Antibacterial activity image of the CSTCF nanocomposites. (A) Shows the green synthesized CSTCF(G) nanocomposite. (B) Shows the chemically synthesized CSTCF(C) nanocomposite. (C) Shows an image of the green synthesized CSTCF(G) nanocomposite against pathogens. (D) Shows an image of the chemically synthesized CSTCF(C) nanocomposite against pathogens. The percentage inhibitions of bacterial growth against the CSTCF(G) and CSTCF(C) nanocomposites are shown in (A) and (B). The data are presented as the mean ± SD of three replications. The data were analysed statistically by one-way analysis of variance followed by Dunnett's multiple range test (Tukey's *post hoc* test) using Graph Pad Prism software. Statistical significance: “*” and “**” represent *p* < 0.05 and *p* < 0.01.

The results reported in Table 3 reveal that the green and chemically synthesized CSTCF(G) and CSTCF(C) nanocomposites were effective against all the tested bacteria. Zone of inhibition images provided in Fig. 12 also show that the results suggested the dose-dependent antibacterial activity of the CSTCF(G) and CSTCF(C) nanocomposites. Similar results were observed by a reviewer report,^101,103^ which reported that a chitosan/starch/AgNP nanocomposite film inhibited the growth of Gram-negative *E. coli* more than Gram-positive *Staphylococcus* sp and *Bacillus* sp.^101,103^

**Table:** Antibacterial activity of CSTCF(G) and CSTCF(C) against several pathogenic bacteria

Pathogen	20 μl ml^−1^	40 μl ml^−1^	60 μl ml^−1^	80 μl ml^−1^
**Green synthesised CSTCF(G) nanocomposite**				
*Staphylococcus* sp	2.3 ± 0.5	5.3 ± 0.5	9.3 ± 0.5	12.1 ± 0.2
*Bacillus* sp	7.5 ± 0.5	10 ± 1.0	13.3 ± 1.5	15 ± 1.0
*Enterobacter*	3.3 ± 1.5	9.6 ± 1.5	12.8 ± 0.7	14.6 ± 0.5
*E. coli*	5.3 ± 2.0	3.6 ± 1.5	8.3 ± 1.5	12 ± 2
*Pseudomonas* sp	5.6 ± 1.5	7.3 ± 1.5	13 ± 2.64	14.6 ± 2.0

**Chemically synthesised CSTCF(C) nanocomposite**				
*Staphylococcus* sp	4.3 ± 1.5	6.3 ± 1.5	8.3 ± 1.5	10.3 ± 1.5
*Bacillus* sp	2 ± 1	3.3 ± 1.5	8.6 ± 1.5	12.3 ± 2.08
*Enterobacter*	5.6 ± 1.5	8 ± 1	12 ± 2	13.3 ± 1.5
*E. coli*	7 ± 2	7 ± 1.7	12 ± 2	16.6 ± 1.5
*Pseudomonas* sp	5.3 ± 1.5	7.3 ± 1.5	9 ± 1	14 ± 2

## 4. Conclusion

The physicochemical profiles and biological functions of green and chemically manufactured CSTCF nanocomposites were successfully compared in this work. The existence and quality of these nanocomposites were verified using XRD, SEM-EDX, UV, and FTIR techniques. Due to the presence of many phyto-constituents, leaf extract from *Salacia reticulata* served as a reducing and capping agent. A different shape was discovered for the CSTCF(C) nanocomposite. Intriguingly, the *Salacia reticulata*, CSTCF(G), and CSTCF(C) nanocomposite dramatically decreased the cell survival dose-dependently in human keratinocyte (HaCaT) and human breast cancer (MCF-7) cells during *in vitro* cytotoxicity evaluation. The green and chemically synthesised particles and extract showed encouraging cytotoxic effects, although the CSTCF(G) nanocomposite and extract had a higher potential for cytotoxicity. The bioactivities of the synthesised CSTCF(G) nanocomposite have been shown in this study to have strong anti-inflammatory and anti-diabetic effects for the significant inhibition of high activation. All the samples of the CSTCF(G) nanocomposite can be used as germ-resistant agents in a variety of drug delivery sectors since they are effective antibacterial compounds. Finally, the created CSTCF nanocomposites and the outcome show that the chemically created nanoparticles were less stable than the naturally created ones. According to this study, compared to chemically manufactured nanocomposites, the CSTCF(G) nanocomposite exhibits superior form and significantly decreased toxicity to zebrafish eggs in plant species that include nanoparticles. Both the green and chemically synthesised CSTCF nanocomposites in the current study show positive anti-diabetic effects, although at concentrations ranging from 10 to 50 μl ml^−1^, the CSTCF(G) nanocomposite outperformed the CSTCF(C) nanocomposite and *Salacia reticulata*. According to the results of the photocatalytic tests, the CSTCF(G) and CSTCF(C) nanocomposites are the samples that are best suited to the photodegradation of malachite green (90% under visible light in 120 min). The synthesised CSTCF(G) nanocomposite has a better potential activity than the CSTCF(C) nanocomposite, as shown by the anti-inflammatory graph. The encouraging findings might provide a risk-free starting point for the use of the green CSTCF(G) nanocomposite in the pharmaceutical sector. Additionally, the current study revealed that green nanoparticle synthesis is more affordable, environmentally friendly, and biocompatible than chemical synthesis.

## Ethics approval

All experiments were carried out in compliance with the standard ethical guidelines and under the control of the Sri Paramakalyani Centre for Excellence in Environmental Science-Manonmaniam Sundaranar University Animal Ethics Committee.

## Consent to participate and consent for publication

Informed consent was obtained from all individual participants included in the study.

## Code availability

Not applicable.

## Data availability

The following data from the manuscript are available from the corresponding author on reasonable request.

## Author contributions

This work was carried out in collaboration among all authors. G. Sabeena designed the study and performed the research. Author S. Vainath Praveen wrote the first draft of the manuscript. Author E. Pushpalakshmi managed the statistical analysis of the study, analyses and revision. The corresponding author G. Annadurai oversaw the above work and devised the experimental work technique. All authors read and approved the final manuscript.

## Conflicts of interest

The authors declare that they have no conflict of interest for this study.

## Supplementary Material
